# Supplementary material for: Sociodemographic influences on children’s out-of-school time organized activity participation and physical activity in rural communities: A cross-sectional study
Source: PLoS One. 2025 May 28;20(5):e0324560. doi: 10.1371/journal.pone.0324560 (PMC12118984; doi:10.1371/journal.pone.0324560)
Supplement: Attachment — Submitted filename: Response to Reviewers 2.docx [file pone.0324560.s002.docx]

Adrian Cyrus Luczon

*PLOS ONE*

October 18, 2024

Dear Adrian,

Re: Edits requested on manuscript reference No. PONE-D-24-23961R1

On behalf of my colleagues and myself, thank you for allowing us to revise our Data Availability statement to align with *PLOS*’ Data Policy by also providing non-author contact information for data access. As a result, we have revised our Data Availability statement to the following:

Data for this and the larger Whole-of-Community Youth Physical Activity (Wellscapes) study are ethically restricted to protect child confidentiality. The Institutional Review Board ([irbora@unmc.edu](mailto:irbora@unmc.edu)) and Sponsored Programs Administration ([spadmin@unmc.edu](mailto:spadmin@unmc.edu)) at University of Nebraska Medical Center forbid making the data publicly available because informed consent from study participants did not cover public deposition of data. However, the minimal data set underlying this study’s findings is stored at the University of Nebraska Medical Center on a secure server and can be accessed by interested researchers on-site pending review of data usage application. Therefore, researchers who meet the criteria for access to confidential data may send their request to the University of Nebraska Medical Center, where the data is housed ([coph@unmc.edu](mailto:coph@unmc.edu)), or submit a request to Wellscapes Primary Investigator and senior author, Dr. David A. Dzewaltowski ([david.dzewaltowski@unmc.edu](mailto:david.dzewaltowski@unmc.edu)).

Additionally, thanks for updating our Data Availability statement on our behalf. We hope that our edits and response satisfactorily address all the issues and concerns you have noted.

Again, thank you for giving us the opportunity to revise our Data Availability statement. We look forward to hearing from you regarding our submission and would be glad to respond to any further questions and comments that you may have.

Sincerely,

Mary J. Von Seggern

Manuscript No. **PONE-D-24-23961R1** – edits requested on **October 17, 2024**

| **Journal Requirements and Prompts** | |
| --- | --- |
| **Comments** | **Author Response and Modification** |
| **1. Journal Requirement – Data Availability**  Thank you for updating your Data Availability statement to:  "Data for this and the larger Whole-of-Community Youth Physical Activity (Wellscapes) study are ethically restricted to protect child confidentiality. The Institutional Review Board (irbora@unmc.edu) and Sponsored Programs Administration (spadmin@unmc.edu) at University of Nebraska Medical Center forbid making the data publicly available because informed consent from study participants did not cover public deposition of data. However, the minimal data set underlying this study’s findings is stored at the University of Nebraska Medical Center on a secure server and can be accessed by interested researchers on-site pending review of data usage application. Data requests should be submitted to Dr. David A. Dzewaltowski (david.dzewaltowski@unmc.edu)."  PLOS’ Data Policy states that authors cannot be the sole named individuals responsible for ensuring data access (https://journals.plos.org/plosone/s/data-availability#loc-unacceptable-data-access-restrictions).  **Therefore, before we can proceed, please also provide non-author contact information for a data access committee, ethics committee, or other institutional body to which data requests may be sent.**  **We will update your Data Availability statement on your behalf to reflect the information you provide.** | 1. Thanks for highlighting the journal’s requirements concerning data availability. Therefore, we have revised the Data Availability statement to also include non-author contact information for data access (and thanks in advance for updating our Data Availability statement based on the information below):   Data for this and the larger Whole-of-Community Youth Physical Activity (Wellscapes) study are ethically restricted to protect child confidentiality. The Institutional Review Board ([irbora@unmc.edu](mailto:irbora@unmc.edu)) and Sponsored Programs Administration ([spadmin@unmc.edu](mailto:spadmin@unmc.edu)) at University of Nebraska Medical Center forbid making the data publicly available because informed consent from study participants did not cover public deposition of data. However, the minimal data set underlying this study’s findings is stored at the University of Nebraska Medical Center on a secure server and can be accessed by interested researchers on-site pending review of data usage application. Therefore, researchers who meet the criteria for access to confidential data may send their request to the University of Nebraska Medical Center, where the data is housed ([coph@unmc.edu](mailto:coph@unmc.edu)), or submit a request to Wellscapes Primary Investigator and senior author, Dr. David A. Dzewaltowski ([david.dzewaltowski@unmc.edu](mailto:david.dzewaltowski@unmc.edu)). |
