# Supplementary material for: Sociodemographic influences on children’s out-of-school time organized activity participation and physical activity in rural communities: A cross-sectional study
Source: PLoS One. 2025 May 28;20(5):e0324560. doi: 10.1371/journal.pone.0324560 (PMC12118984; doi:10.1371/journal.pone.0324560)
Supplement: Attachment — Submitted filename: Response to Reviewers.docx [file pone.0324560.s003.docx]

Catherine M. Capio, Ph.D.

Academic Editor

*PLOS ONE*

January 27, 2025

Dear Dr. Capio,

Re: Resubmission of manuscript reference No. PONE-D-24-23961R1

On behalf of my colleagues and myself, thank you for inviting us to address some remaining concerns and submit a revised version of our manuscript, now titled, “Sociodemographic influences on children’s out-of-school time organized activity participation and physical activity in rural communities: A cross-sectional study,” to *PLOS ONE*. We also appreciate the time and effort you and each of the reviewers have dedicated to providing additional feedback on ways to improve the dissemination of knowledge through our manuscript. As a result, we have incorporated changes in the revised manuscript that reflect the comments you and the reviewers have provided in your letter dated December 16, 2024. Similar to our previous revision, we have attempted to explain changes made in reaction to all comments in a point-by-point fashion displayed in a table format below, corresponding with the letter’s organization (i.e., Journal Requirements, Additional Editor Comments, and Reviewers’ Comments), and hope that our edits and the responses we have provided below satisfactorily address all the remaining concerns you and the reviewers have noted.

Again, thank you for giving us the opportunity to strengthen our manuscript with your valuable feedback. We look forward to hearing from you regarding our submission and would be glad to respond to any further questions and comments that you and the reviewers may have.

Sincerely,

Mary J. Von Seggern

Manuscript No. **PONE-D-24-23961R1** – revision required by **January 30, 2025**

| **Part I – Journal Requirements** | |
| --- | --- |
| **Comments** | **Author Response and Modification** |
| **1. Journal Requirements – Reference List**  **Please review your reference list to ensure that it is complete and correct**. If you have cited papers that have been retracted, please include the rationale for doing so in the manuscript text, or remove these references and replace them with relevant current references. **Any changes to the reference list should be mentioned in the rebuttal letter that accompanies your revised manuscript**. If you need to cite a retracted article, indicate the article’s retracted status in the References list and also include a citation and full reference for the retraction notice. | 1. Thank you for bringing this to our attention, and we have reviewed our reference list to ensure it is complete and correct, including based on the changes listed below (in which a total of 7 references were completely removed from the paper and 5 references were added):      1.1 We removed references 76-78 (Belcher et al., 2010; Change et al., 2019; and Whitt-Glover et al., 2009) on pg. 22, line 440, since we revised this paragraph based on Reviewer #1’s feedback of discussing findings in relation to the ecological theories presented in the beginning; although, the *Whitt-Glover et al., 2009 citation was used later in the paper (so it is still in the reference list)*. 1.2 Additionally, we removed reference 79 (Probst et al., 2018) on pg. 22, line 446, and reference 80 (Yousefian et al. 2009) on pg. 22, line 453, since we are no longer comparing rural children to urban peers (and focusing on differences within rural community systems and connecting the findings back to ecological theories presented in the beginning). 1.3 To further address Reviewer comments in the Discussion section, primarily related to the unacknowledged shift from OST organized activities to afterschool programs, as well as the need to address the practical significance of the study, we removed afterschool references, specifically reference 82 (Trost et al., 2008) on pg. 23, line 480 and reference 83 (Mears and Jago, 2016) on pg. 24, line 488. Although reference 84 (Weaver et al., 2015) was deleted on pg. 24, line 490, *it was not fully removed from the article* as it was cited on pg. 24, line 496. 1.4 To support the practical significance of the study, we added three references, specifically: (1) Piercy et al., 2018; (2) Piercy and Troiano, 2018; and (3) Sriram et al., 2021, on pg. 23, lines 477-478. 1.5 We also added two citations, Barker and Schoggen, 1973, and Gump and Adelberg, 1978, on pg. 26, line 544, to support one of our study’s finding related to family income and weekday and weekend OST MVPA. In response to this change (and emphasis on Barker and colleagues’ work to support the finding), we removed reference 96 (Ludden, 2011) and reference 25 (Ferris et al., 2013), but *the latter reference remains included in the article* from previous citations. |
| **Part II – Additional Editor Comments** | |
| **Comments** | **Author Response and Modification** |
| **Editor: General Comments**  Thank you for your revised manuscript. While the points raised in the original submission were generally addressed, there are a few outstanding concerns that the authors should consider. | Thank you for taking time to review and provide comments on our revised manuscript. We appreciate your feedback, as well as the opportunity to revise and resubmit the manuscript once again. |
| **Editor: Particular Comments**  1. I also note that the comment from Reviewer #2 from the previous version might have been a bit misunderstood; consider re-thinking the revision related to this. | 1. Thank you for this feedback, and we realize the comment from Reviewer #2 from the previous version was misunderstood and have revised the Discussion to include a specific statement related to the study’s practical significance as follows:   *Since many (>70%) children in the U.S. are not physically active at the recommended levels [7,8], calls for any increase in childhood PA have been issued, as evidence suggests even small amounts or short episodes of PA provide health benefits both now and in the future (e.g., reduced anxiety symptoms and improved quality of life, respectively) [Piercy et al. 2018; Piercy and Troiano 2018; Sriram et al. 2021]. Therefore, based on the results of our study and others examining the PA patterns of children attending OST structured settings, like afterschool programs, if clubs and other organized activities can schedule time for PA within setting routines, this may positively influence PA promotion efforts* (Pages 23-24, lines 473-493)  We also added a comment in the Conclusions section about the practical significance as follows:  *From a public health perspective, advocates acknowledge “some physical activity is better than none” [Piercy and Troiano 2018]; therefore, it might be worthwhile for the U.S. to expand the National Youth Sports Strategy [U.S. Department of Health and Human Services 2019] to include non-sport organized activities.* (Page 31, lines 641-644)   2. We also revised the Conclusions section based on both Reviewers’ feedback (highlighted below) and addressed all other comments accordingly. |
| **Part III – Reviewers’ Comments** | |
| **Comments** | **Author Response and Modification** |
| **Reviewer #1: General Comments**  Thank you for the opportunity to review this revised manuscript. I would like to commend the authors for a structured and thoroughly conducted revision. I believe most of my previous comments have been addressed, and in many cases, clarified effectively. I have a few further comments on this revised version. | Thank you for taking time to review and provide comments on our manuscript once again. We greatly appreciate your additional feedback and have addressed all comments presented. Thanks again. |
| **Reviewer #1: Specific Comments – Overall**  1. Use of Theory: Barker/Bronfenbrenner is employed as a form of justification for the study – a purpose I find less convincing unless the authors directly tie back to this framework later in the paper. In the discussion, other, more loosely connected explanatory models are introduced instead. I would still suggest that the findings be discussed in relation to the theories highlighted in the introduction. If not, it may be better to remove these theoretical references from the introduction altogether to avoid confusion. | 1. We appreciate your feedback and have more directly tied the study’s findings back to the ecological theories mentioned in the beginning of the manuscript as follows:  1.1 In the opening paragraph of the Discussion, highlighting the key finding that approximately 70% of children reported participating in an OST organized activity, we suggested this finding may be related to Barker’s Manning Theory and specifically added the following:  Thus, *the effects of underpopulation (or “undermanning”) witnessed by Barker and Gump in 1964 may still be relevant today* given the high prevalence of participation among rural children and adolescents in these settings *[Barker and Gump 1964]*. *Even if rural children are pressured to participate in OST organized activities out of necessity, participation can significantly impact childhood development, including the improvement of population health PA outcomes* [38,71]. (Pages 20-21, lines 410-416)  1.2 We also revised the third paragraph of the Discussion to shift the focus back to the ecological theories mentioned in the beginning of the paper. The revision includes the following:  Our work also revealed no differences in rural children’s organized activity participation classified by family income. *Although this finding may not be surprising in communities where “undermanning” exists and settings cannot afford to intentionally (or unintentionally) exclude children from participating, it is noteworthy since youth sport settings have been shown to marginalize children living in rural communities based on social classifications, including those from low-income families [Kellstedt et al. 2021; Von Seggern et al. 2024].* For example, Kellstedt et al. [46] found that FRLS was a significant (p < 0.05) predictor of youth sport participation, in which *rural* children from full pay families demonstrated almost four times higher participation rates in youth sport compared to those from FRLS families (OR = 3.91, 95% CI = 1.95-7.8) [46]. This suggests that OST non-sport organized activities may be more financially accessible than OST organized youth sport opportunities, particularly for economically disadvantaged children *living in rural communities. Cost, however, is not the only factor influencing participation, and within rural communities, non-sport organized activities may be more responsive to different levels of environmental influences on children’s behavior [Bronfenbrenner 1979], such as parental or guardian need for childcare services after the school day (e.g., school-based afterschool programs) if they are commuting out-of-town for employment purposes or their employer does not offer flexible working hour or work-from-home options. Thus, it is necessary to account for different factors influencing OST organized activity participation, including who (e.g., parents, friends, and teachers) and what (e.g., availability, time constraints, and past experiences) are driving children’s participation in these specific behavior settings.* This information can uncover why children frequent specific community OST behavior settings within a given social context and could help identify ways to reverse or remove participation barriers and support more equitable organized activity opportunities where all children are encouraged to participate. (Pages 21-22, lines 433-455)  1.3 We also attempted to more explicitly connect social structural differences highlighted in the Discussion to Bronfenbrenner’s ecology theory as follows:  Social structural differences, *found within all layers of Bronfenbrenner’s ecological systems theory from the immediate microsystem to the wider macrosystem [Bronfenbrenner 1979], including broader societal* gender norms influencing types of opportunities, roles within opportunities, and leader-level practices, may also help to explain sex-based PA disparities [54,88]. (Page 25, lines 512-515)  1.4 In another paragraph in the Discussion, we provided another explanation of one of the study’s findings in relation to Barker and colleagues’ work on behavior settings as follows:  Another possible explanation, *consistent with Barker and colleagues’ work on behavior settings, is that in often “undermanned” rural communities, children from lower-income families may feel obligated to participate in OST organized activities out of necessity or a desire for community belonging [Barker and Gump 1964; Barker and Schoggen 1973; Gump and Adelberg, 1978]; in turn, accumulating similar amounts of weekday and weekend OST MVPA with their higher-income participating peers*. (Page 26, lines 538-546) |
| **Reviewer #1: Specific Comments – Results/Discussion**  2. The presentation of results related to the contribution of OST activities to PA is somewhat confusing. You found no significant differences between participants and non-participants in organised OST activities, yet you suggest (p. 22, line 450) that these activities can still be beneficial for PA. This appears contradictory and should be reviewed. | 2. We have revised the aforementioned paragraph in the Discussion to zoom in on why these activities can still be beneficial for PA, particularly, since we found that children in our study who participated in afterschool programs reported significantly more daily minutes of OST MVPA per weekday than those who did not participate (as presented in the Results section on pg. 16, lines 340-344, and in Fig. 1). As a result, this finding prompted the “unacknowledged shift from OST organized activities to afterschool activities” mentioned in a later comment, but we have attempted to clean this up while making a case for why non-sport OST organized activities can still be beneficial for PA, where we acknowledge any PA is better than no PA (related to Reviewer #2’s first comment). Overall, we combined paragraphs four and five in the Discussion as follows:  Although we found that children who participated in any OST organized activity were not significantly more active during OST on weekdays and weekends than nonparticipants, encouraging participation may still be beneficial given the contribution some OST organized activities have on *children’s* PA, *particularly when participating* [81]. *For instance, children in the present study who participated in afterschool programs reported significantly more daily minutes of OST MVPA per weekday (meandiff = 3.0 ± 1.2 min/day) than those who did not participate in an afterschool program (p = 0.014). Yet, there was no difference in weekend MVPA between program participants and non-participants, suggesting that afterschool programs may provide a meaningful contribution to PA, but only when children attend. Similarly*, a systematic review and meta-analysis by Tassitano et al. [81] found that OST structured settings (i.e., afterschool programs and summer camps) provided youth (aged 3-18 years) with greater amounts of PA during attendance when compared to childcare and school, specifically, 11.7 min/hour and 6.4 min/hour, respectively. *Since many (>70%) children in the U.S. are not physically active at the recommended levels [7,8], calls for any increase in childhood PA have been issued, as evidence suggests even small amounts or short episodes of PA provide health benefits both now and in the future (e.g., reduced anxiety symptoms and improved quality of life, respectively) [Piercy et al. 2018; Piercy and Troiano 2018; Sriram et al. 2021].* Therefore, based on the results of our study and others examining the PA patterns of children attending *OST structured settings, like afterschool programs,* if clubs and other organized activities can schedule time for PA within setting routines, this may positively influence PA promotion efforts. However, scholars warn simply allocating more time in the schedule for PA opportunities may not be sufficient for increasing children’s MVPA. Specifically, the type of PA is also critical (e.g., structured vs. unstructured, individual vs. group) [84], especially in rural communities where evidence indicates non-sport organized activities are less structured and less supervised [25,38]. Thus, inserting shorter bouts of more structured PA opportunities into these settings may be more advantageous and practical for both rural children and adult leaders*.* (Pages 23-24, lines 461-493) |
| **Reviewer #1: Specific Comments – Results/Discussion**  3. In the discussion, there is an unacknowledged shift from OST organised activities to afterschool activities. If you intend to discuss afterschool programmes and present findings specific to them, the relationship between these types of activities should be described and defined more clearly earlier in the paper. While this is addressed to some extent, I believe clearer definitions would aid an international readership in understanding the results. Additionally, the rationale for this shift in focus should be clarified – I suggest explaining why the discussion transitions from OST in general to afterschool programmes specifically. | 3. Per our previous response (to Specific Comment #2.), we revised the section transitioning from any OST PA to afterschool program PA, including the removal of several sentences and citations specifically focused on afterschool programming PA. However, we remain committed to zooming in on afterschool programs given children in the present study who participated in afterschool programs reported significantly more daily minutes of OST MVPA per weekday than non-participants. Therefore, if other settings can insert time devoted to PA, like afterschool programs, this may positively influence PA promotion efforts (where any PA is better than no PA). The section was revised as follows:  Although we found that children who participated in any OST organized activity were not significantly more active during OST on weekdays and weekends than nonparticipants, encouraging participation may still be beneficial given the contribution some OST organized activities have on *children’s* PA, *particularly when participating* [81]. *For instance, children in the present study who participated in afterschool programs reported significantly more daily minutes of OST MVPA per weekday (meandiff = 3.0 ± 1.2 min/day) than those who did not participate in an afterschool program (p = 0.014). Yet, there was no difference in weekend MVPA between program participants and non-participants, suggesting that afterschool programs may provide a meaningful contribution to PA, but only when children attend. Similarly*, a systematic review and meta-analysis by Tassitano et al. [81] found that OST structured settings (i.e., afterschool programs and summer camps) provided youth (aged 3-18 years) with greater amounts of PA during attendance when compared to childcare and school, specifically, 11.7 min/hour and 6.4 min/hour, respectively. *Since many (>70%) children in the U.S. are not physically active at the recommended levels [7,8], calls for any increase in childhood PA have been issued, as evidence suggests even small amounts or short episodes of PA provide health benefits both now and in the future (e.g., reduced anxiety symptoms and improved quality of life, respectively) [Piercy et al. 2018; Piercy and Troiano 2018; Sriram et al. 2021].* Therefore, based on the results of our study and others examining the PA patterns of children attending *OST structured settings, like afterschool programs,* if clubs and other organized activities can schedule time for PA within setting routines, this may positively influence PA promotion efforts. However, scholars warn simply allocating more time in the schedule for PA opportunities may not be sufficient for increasing children’s MVPA. Specifically, the type of PA is also critical (e.g., structured vs. unstructured, individual vs. group) [84], especially in rural communities where evidence indicates non-sport organized activities are less structured and less supervised [25,38]. Thus, inserting shorter bouts of more structured PA opportunities into these settings may be more advantageous and practical for both rural children and adult leaders*.* (Pages 23-24, lines 461-493) |
| **Reviewer #1: Specific Comments – Conclusions**  4. I believe the Conclusions section would benefit from further revision to contextualise the study’s key findings. If the authors do not wish to tie back to the theoretical framework, the key findings should at least be highlighted more clearly. Currently, much of the Conclusions section consists of repetition. | 4. We appreciate this feedback, as it was also mentioned by Reviewer #2 (Specific Comment #2.). Therefore, we have revised the Conclusions section to primarily highlight the key findings as follows:  *Despite* the complexity of *rural* communities with diverse wellness landscapes *of adult-organized behavior settings*, research on *children’s out-of-school time (OST)* organized activity involvement and subsequent *physical activity (PA)* behaviors has primarily focused on youth sport participation. *Thus,* *the present* study *sought to examine the* sociodemographic factors related to non-sport organized activity participation and how these factors interact to influence OST PA among children living in rural U.S. communities. *While the current results do not support significant OST weekday and weekend PA benefits related to overall OST organized activity participation, several findings suggest these settings should not be overlooked in PA-promotion efforts. Given the prospective reach among children in rural communities, coupled with the fact that family income was not a significant driver of participation in this study, non-sport OST organized activities may be more accessible and responsive to different levels of environmental influences on children’s behavior compared to organized youth sports. Furthermore, if clubs and other organized activities can devote time to PA within setting routines, like afterschool programs, this may positively influence the PA and population health outcomes of children. From a public health perspective, advocates acknowledge “some physical activity is better than none” [Piercy and Troiano 2018]; therefore, it might be worthwhile for the U.S. to expand the National Youth Sports Strategy [U.S. Department of Health and Human Services 2019] to include non-sport organized activities.* (Pages 30-31, lines 618-663) |
| **Reviewer #1: Minor Comments – References**  5. Reference formatting does not consistently follow the Vancouver style throughout the text.  I hope my comments are seen as constructive and can help to further develop and clarify the important contributions of the article. | 5. Thanks for bringing this to our attention, and we have revised our reference formatting to consistently follow the Vancouver style throughout the text. Additionally, we appreciate your comments and believe they have contributed to a stronger, more succinct manuscript. Thank you.  The reference formatting changes are as follows:  5.1 Removed *(2013)* after *Moore and colleagues*, and replaced it with *Moore et al.* and the numerical citation in brackets*.* (Page 3, lines 91-92)  5.2 Removed *(2021)* and *(2024)* after *Kellstedt and colleagues* and *Von Seggern et al.*, respectively, and replaced with *Kellstedt et al.* [*numerical citation*] and *Von Seggern et al.* [*numerical citation*]. (Page 5, lines 135-136)  5.3 Removed *(2018)* after *National AfterSchool Association’s*, and replaced it with [*numerical citation*]. (Page 6, line 152)  5.4 Removed *(2019)* after *Sliwa et al.*, and replaced it with [*numerical citation*]. (Page 6, line 153)  5.5 Removed *(2021)* after *Welk and colleagues*, and replaced it with *Welk et al.* and the numerical citation in brackets*.* (Page 12, lines 287-288)  5.6 Removed *(2015)* after *Sharp and colleagues*, and replaced it with *Sharp et al.* and the numerical citation in brackets*.* (Page 20, lines 401-402)  5.7 Removed *(2008)* after *Guèvremont et al.*, and replaced it with [*numerical citation*]. (Page 21, line 420)  5.8 Removed (*2021)* after *Kellstedt et al.*, and replaced it with [*numerical citation*]. (Page 22, line 440)  5.9 Removed (*2020)* after *Tassitano and colleagues*, and replaced it with *Tassitano et al.* and the numerical citation in brackets*.* (Page 23, lines 470-471)  5.10 Removed (*2008)* after *Voss et al.*, and replaced it with [*numerical citation*]. (Page 25, line 526)  5.11 Removed (*2015)* after *Cottrell et al.*, and replaced it with [*numerical citation*]. (Page 26, line 529)  5.12  Removed (*2017)* after *Hankonen and colleagues’*, and replaced it with *Hankonen et al.’s* and the numerical citation in brackets*.* (Page 28, line 574)  5.13 Removed (*2021)* after *Schenkelberg et al.*, and replaced it with [*numerical citation*]. (Page 29, line 597) |
| **Comments** | **Author Response and Modification** |
| **Reviewer #2: General Comments**  Thank you for the opportunity to review this paper again. It would be helpful if the authors could refer to the comments in their response. | Thank you for taking time to review and provide comments on our manuscript once again. We greatly appreciate your additional feedback and have referred to and addressed all comments presented. Thanks again. |
| **Reviewer #2: Specific Comments**  1. While the authors have addressed most my concerns, I believe my original comment #5 was not fully addressed. My question was not about the significance of this study but the practical significance of the study. While the observed group level differences were statistically significant, the differences were 3 minutes over a half day on weekdays or less than 10 minute over a whole day. Are these differences meaningful in terms of the health impact? how do the authors interpret the practical significance?  **Original comment #5 -** While some statistically significant differences were observed in this study, these differences were 3 minutes over a half day on weekdays or less than 10 minutes over a whole day – which may be less meaningful in terms of the health impact. How would the authors interpret the clinical significance of their findings? | 1. Thanks for this feedback, and we realize your previous comment (comment #5 pasted into this document) was misunderstood. We have added a specific statement in the Discussion section, after mentioning the 3-minute difference, which addresses the practical significance of the study (based on the scientific rationale informing the development of the nation’s PA guidelines) as follows:   1.1 *Since many (>70%) children in the U.S. are not physically active at the recommended levels [7,8], calls for any increase in childhood PA have been issued, as evidence suggests even small amounts or short episodes of PA provide health benefits both now and in the future (e.g., reduced anxiety symptoms and improved quality of life, respectively) [Piercy et al. 2018; Piercy and Troiano 2018; Sriram et al. 2021]. Therefore, based on the results of our study and others examining the PA patterns of children attending OST structured settings, like afterschool programs, if clubs and other organized activities can schedule time for PA within setting routines, this may positively influence PA promotion efforts* (Pages 23-24, lines 473-493)  1.2 We also added a comment in the Conclusions section about the practical significance as follows:  *From a public health perspective, advocates acknowledge “some physical activity is better than none” [Piercy and Troiano 2018]; therefore, it might be worthwhile for the U.S. to expand the National Youth Sports Strategy [U.S. Department of Health and Human Services 2019] to include non-sport organized activities.* (Page 31, lines 641-644) |
| **Reviewer #2: Specific Comments**  2. The revised conclusion section is too lengthy. I recommend transferring some of the content to the discussion section. | 2. We appreciate this feedback, as it was also mentioned by Reviewer #1 (Specific Comment #4.). Therefore, we have transferred some of the content in the Conclusions section to the Discussion section as follows:  2.1 In the third paragraph of the Discussion, we added information about parental/guardian need for childcare before or after the school day as follows:  Cost, however, is not the *factor influencing participation, and within rural communities, non-sport organized activities may be more responsive to different levels of environmental influences on children’s behavior [Bronfenbrenner 1979], such as parental or guardian need for childcare services after the school day (e.g., school-based afterschool programs) if they are commuting out-of-town for employment purposes or their employer does not offer flexible working hour or work-from-home options*. (Page 22, Lines 446-452)  2.2 We also condensed the Conclusions section by focusing on the study’s key findings and overall practical significance as follows:  *Despite* the complexity of *rural* communities with diverse wellness landscapes *of adult-organized behavior settings*, research on *children’s out-of-school time (OST)* organized activity involvement and subsequent *physical activity (PA)* behaviors has primarily focused on youth sport participation. *Thus,* *the present* study *sought to examine the* sociodemographic factors related to non-sport organized activity participation and how these factors interact to influence OST PA among children living in rural U.S. communities. *While the current results do not support significant OST weekday and weekend PA benefits related to overall OST organized activity participation, several findings suggest these settings should not be overlooked in PA-promotion efforts. Given the prospective reach among children in rural communities, coupled with the fact that family income was not a significant driver of participation in this study, non-sport OST organized activities may be more accessible and responsive to different levels of environmental influences on children’s behavior compared to organized youth sports. Furthermore, if clubs and other organized activities can devote time to PA within setting routines, like afterschool programs, this may positively influence the PA and population health outcomes of children. From a public health perspective, advocates acknowledge “some physical activity is better than none” [Piercy and Troiano 2018]; therefore, it might be worthwhile for the U.S. to expand the National Youth Sports Strategy [U.S. Department of Health and Human Services 2019] to include non-sport organized activities.* (Pages 30-31, lines 618-663) |
