# Supplementary material for: Sociodemographic influences on children’s out-of-school time organized activity participation and physical activity in rural communities: A cross-sectional study
Source: PLoS One. 2025 May 28;20(5):e0324560. doi: 10.1371/journal.pone.0324560 (PMC12118984; doi:10.1371/journal.pone.0324560)
Supplement: Attachment — Submitted filename: Response to Reviewers_R3.docx [file pone.0324560.s004.docx]

Catherine M. Capio, Ph.D.

Academic Editor

*PLOS ONE*

April 24, 2025

Dear Dr. Capio,

Re: Resubmission of manuscript reference No. PONE-D-24-23961R2

On behalf of my colleagues and myself, thank you for inviting us to address the remaining concern related to the conclusion and submit a revised version of our manuscript titled, “Sociodemographic influences on children’s out-of-school time organized activity participation and physical activity in rural communities: A cross-sectional study,” to *PLOS ONE*. We also appreciate the time and effort you and each of the reviewers have dedicated to providing additional feedback on ways to improve our manuscript. As a result, we have incorporated changes in the revised manuscript that reflect the comments you and the reviewers have provided in your letter dated March 19, 2025. Similar to our previous revisions, we have attempted to explain changes made in reaction to all comments in a point-by-point fashion displayed in a table format below, corresponding with the letter’s organization (i.e., Journal Requirements, Additional Editor Comments, and Reviewers’ Comments), and hope that our edits and the responses we have provided below satisfactorily address all the remaining concerns you and the reviewers have noted.

Again, thank you for giving us the opportunity to strengthen our manuscript with your valuable feedback. We look forward to hearing from you regarding our submission and would be glad to respond to any further questions and comments that you and the reviewers may have.

Sincerely,

Mary J. Von Seggern

Manuscript No. **PONE-D-24-23961R2** – revision required by **May 03, 2025**

| **Part I – Journal Requirements** | |
| --- | --- |
| **Comments** | **Author Response and Modification** |
| **1. Journal Requirements – Reference List**  **Please review your reference list to ensure that it is complete and correct**. If you have cited papers that have been retracted, please include the rationale for doing so in the manuscript text, or remove these references and replace them with relevant current references. **Any changes to the reference list should be mentioned in the rebuttal letter that accompanies your revised manuscript**. If you need to cite a retracted article, indicate the article’s retracted status in the References list and also include a citation and full reference for the retraction notice. | 1. Thank you for bringing this to our attention, and we have reviewed our reference list to ensure it is complete and correct. Additionally, no changes were made to our reference list during this revision process. |
| **Part II – Additional Editor Comments** | |
| **Comments** | **Author Response and Modification** |
| **Editor: General Comments**  Thank you for submitting your manuscript to PLOS ONE. The revised manuscript addressed most of the reviewers' concerns, save for one remaining point related to the conclusion. We invite you to address this remaining point that was raised by both reviewers, and submit a revised version of the manuscript. | Thank you for taking time to review and provide comments on our revised manuscript. We appreciate your feedback, as well as the opportunity to revise and resubmit the manuscript once again. |
| **Editor: Particular Comments**  1. Thank you for the revised manuscript, which now addresses nearly all of the comments raised by the reviewers. One last point that needs to be addressed relates to your conclusions, which was noted by both reviewers. If you could please have a look at their comments and improve the conclusion, that would be great. | Thank you for this feedback, and we revised our conclusions based on each reviewer’s feedback as highlighted below:  Reviewer #1 –  1. We addressed Reviewer #1’s comment by acknowledging the limitations of the cross-sectional study design, in which a causal link between participation in after-school activities and MVPA cannot be established. Thus, we added this information to the Limitations section as follows:  *Furthermore, we acknowledge the limitation of the cross-sectional study design, in which a causal link between participation in OST non-sport organized activities, including daily afterschool programs, and OST MVPA cannot be established*. (Page 28, lines 575-577)  Reviewer #2 –  2. We addressed Reviewer #2’s comment by revising the Conclusions section to enhance clarity and impact as follows:  *The present study aimed to examine the sociodemographic factors related to out-of-school time (OST) non-sport organized activity participation and how these factors interact to influence OST physical activity (PA) among children living in rural U.S. communities. The findings suggest that, along with other factors, overall OST non-sport organized activity participation does not play a critical role in maximizing the amount of PA that rural children engage in during OST on weekdays and weekends. Yet, these settings should not be overlooked in PA-promotion efforts provided the fairly high percentage (70%) of rural children participating in non-sport organized activities, coupled with the fact that grade and family income were not a significant driver of participation in this study. Thus, non-sport OST organized activities may be more accessible and responsive to different levels of environmental influences on rural children’s behavior, particularly when considering the well-documented barriers to organized youth sport participation, like cost. More research is needed to explore how these activities could be effectively designed to increase PA since “some physical activity is better than none” [79] and afterschool programs, which often devote time to PA within daily setting routines, can provide a blueprint to consider. Therefore, increasing participation and opportunities for PA within non-sport organized activities could positively impact the MVPA and population health outcomes of children living in rural communities, and advocating for the advancement of an updated National Youth Sports Strategy [42] to include non-sport organized activities may aid in ensuring children nationwide have the opportunity to achieve optimal health*. (Pages 28-30, lines 593-622)  3. Additionally, we revised the last sentence in the abstract to improve the conclusion (per previous changes made in the Conclusions section) as follows:  *Designing OST organized activity settings to be more accessible and include opportunities for PA may help ensure children can achieve optimal health*. (Page 2, lines 61-63) |
| **Part III – Reviewers’ Comments** | |
| **Comments** | **Author Response and Modification** |
| **Reviewer #1: General Comments**  Thank you for the opportunity to review your manuscript once again. I believe the authors have done a great job addressing and responding to the comments I had on the previous version. It is a thorough piece of work, and they deserve recognition for that.  Best of luck with the revision, and I look forward to seeing the paper published. | Thank you for taking time to review our manuscript once again. We greatly appreciate your feedback, well wishes, and additional comments to help strengthen this piece of work. Thanks again. |
| **Reviewer #1: Specific Comments**  1. The only comment I feel has not been fully addressed is comment 2, the one that concerns the conclusions regarding the contribution of after-school activities to MVPA. Since this is cross-sectional data, there is no evidence that after-school activities have contributed to MVPA rather than the other way around. I believe this should at least be mentioned somewhere in the discussion, preferably in the limitations section.  **Original Comment #2** - The presentation of results related to the contribution of OST activities to PA is somewhat confusing. You found no significant differences between participants and non-participants in organised OST activities, yet you suggest (p. 22, line 450) that these activities can still be beneficial for PA. This appears contradictory and should be reviewed. | 1. We appreciate your feedback and have more directly addressed this comment by acknowledging the limitations of the cross-sectional study design, in which a causal link between participation in after-school activities and MVPA cannot be established. Thus, we added this information to the Limitations section as follows:  *Furthermore, we acknowledge the limitation of the cross-sectional study design, in which a causal link between participation in OST non-sport organized activities, including daily afterschool programs, and OST MVPA cannot be established*. (Page 28, lines 575-577) |
| **Comments** | **Author Response and Modification** |
| **Reviewer #2: General Comments**  Thank you for revising the manuscript. | Thank you for taking time to review and provide comments on our manuscript once again. We greatly appreciate your additional feedback to enhance the clarity and impact of our work. Thanks again. |
| **Reviewer #2: Specific Comments**  1. I believe the conclusion could be more concise to enhance clarity and impact. | 1. We appreciate this feedback, as Reviewer #1 and the Editor also mentioned it. Therefore, we have revised our Conclusions section as follows:  *The present study aimed to examine the sociodemographic factors related to out-of-school time (OST) non-sport organized activity participation and how these factors interact to influence OST physical activity (PA) among children living in rural U.S. communities. The findings suggest that, along with other factors, overall OST non-sport organized activity participation does not play a critical role in maximizing the amount of PA that rural children engage in during OST on weekdays and weekends. Yet, these settings should not be overlooked in PA-promotion efforts provided the fairly high percentage (70%) of rural children participating in non-sport organized activities, coupled with the fact that grade and family income were not a significant driver of participation in this study. Thus, non-sport OST organized activities may be more accessible and responsive to different levels of environmental influences on rural children’s behavior, particularly when considering the well-documented barriers to organized youth sport participation, like cost. More research is needed to explore how these activities could be effectively designed to increase PA since “some physical activity is better than none” [79] and afterschool programs, which often devote time to PA within daily setting routines, can provide a blueprint to consider. Therefore, increasing participation and opportunities for PA within non-sport organized activities could positively impact the MVPA and population health outcomes of children living in rural communities, and advocating for the advancement of an updated National Youth Sports Strategy [42] to include non-sport organized activities may aid in ensuring children nationwide have the opportunity to achieve optimal health*. (Pages 28-30, lines 593-622) |
